# Notes and News

**Published:** 1889-02-09

**Authors:** 


### Notes and News.

Collected and Communicated.

A great exhibition will be held at York from June to October, partly in aid of the County Hospital, partly in aid of the York Dispensary. There will be a special sanitary section, and in the scientific and chemical section, pharmaceutical articles will find a place.

Ten children have undergone innoculation for rabies at the Pasteur Institute, Paris. They were all bitten by a dog, (presumably mad), at St. Helens, Lancashire, two weeks ago. Unluckily the dog was killed, but Dr. Barron, of Liverpool, has injected some of the virus into a rabbit, hence it is hoped to obtain proof as to whether the dog was mad or not.

Annual meetings were numerous in London last week. First there was that of the Metropolitan Hospital, with Mr. Joseph Fry in the chair. The report was fairly satisfactory, but help is wanted to furnish an additional 100 beds. Then the Royal Maternity Charity had a tale to tell of over three thousand poor women aided; and the Metropolitan Convalescent Institution reported four thousand convalescents given change of air. The Finsbury Dispensary held its 179th annual meeting, a fact which is eloquent testimony to its worth.

A most interesting Christmas gathering took place at Chalmers Hospital on the evening of January 10th. Those of the patients who were able, assembled in the spacious board-room at half-past seven, and were entertained to a selection of views on the magic lantern, which was cleverly manipulated by Mr. Rae, artist, Bridge Street, and varied by singing and playing by the matron and staff of nurses. After a service of fruit, the evening's entertainment was concluded by the patients joining in the hymn "Abide with Me." Christmas Day itself was observed in the hospital by the usual Christmas dinner. The wards were prettily decorated by the nurses, and Christmas cards were given to each patient—gifts of the Pillow Mission and friends in town as usual. The front hall and staircase were tastefully decorated by Mr. Andrew Duncan, the house steward.

On January 26th the annual meeting of the Italian Hospital was held at Queen Square, Bloomsbury, under the presidency of Commendatore T. Catalini. The committee's annual report, which was adopted by the meeting, stated that during the year 1888, 226 in-patients had been relieved; also 2,421 out-patients (of which Italians formed by far the largest nationality). Since its establishment in 1884 the total number of patients relieved had been 10,388 out and 1,063 in-patients. The benefits of the hospital are supplied gratuitously. The institution contains 20 beds—12 female and eight male, but, owing to the pressure on its space, it has been found that the premises are not large enough, and steps are being taken to build an additional ward at a cost of £500; and it is intended to ask the help of the Corporation towards this much-needed extension.

Professor E. J. Bell's lecture at the Sanitary Institute last week on the "Worm Parasites of Human Food" was exceedingly interesting. The lecturer stated that food of all kinds, even onions, sugar-cane, or hens' eggs, may harbour worm parasites of various kinds, but it is not always the case that these parasites are dangerous to man, some dying on entering the body, while others were killed when the food which contained them is sufficiently cooked. When science had worked out the course of the life history of parasites, sanitarians would be equipped with the knowledge which would enable them to cope with their foes.

Our children are still dying in London at the rate of nearly one hundred a week from measles, but still the Asylums Board will not come to the rescue and offer its aid in staying the epidemic. Perhaps instead of "will not," we should say "cannot," only that we think the Board is responsible for the red tape which hampers its movements. Measles are especially prevalent in Battersea, and the evil is, that little sufferers get admitted for accidents into the wards of general hospitals, and give much trouble and cost to managers. It is no unusual thing to see a children's ward placarded just now, "Closed, on account of measles."

Of course the Muswell Hill outrage has interested everyone, and we have all felt sympathy for Mr. George Atkin. But how necessary that sympathy was we never fully comprehended till the following paragraph appeared in the *Derby Daily Telegraph*: "Sir William MacCormac and other magistrates to-day successfully extracted a bullet from the chest of Mr. Geo. Atkin, the victim of the Muswell Hill outrage." With all due regard for magistrates, we should not like to have a body of them as assistants at a surgical operation. Perhaps Sir William was glad to avail himself of their services, and will inform us whether he or the magistrates extracted the bullet?

The annual general meeting of the Royal Ear Hospital, Frith Street, Soho, was held in the hospital on Tuesday, the 15th ult., Mr. John Carr, J.P., treasurer, in the chair. The secretary reported that during the year just closed there were 8,391 out-patient attendances; that 94 patients had been admitted into the in-patient wards; and that these numbers were by far the largest on record since the foundation of the institution in 1816. The financial report showed that, like most other hospitals, there had been a falling-off in subscriptions and donations; and that had it not been for the receipt of a legacy apportioned by the late Earl of Shaftesbury, there would have been a serious deficit at the end of the year. Increased subscriptions and donations are urgently needed to meet the ever-increasing demands on this old-established and most useful charity. A vote of thanks to the surgeons, treasurer, secretary, and matron brought the proceedings to a close.

Miss Soames has had the courage to speak at Brighton on a difficult ethical question. The Hospital for Women and Children has, so far, never admitted unmarried women to its special wards, and Miss Soames pleads for this extension. It must be remembered that Queen Charlotte's admits unmarried women, and that the Queen is patron of that hospital. Miss Soames asked her hearers to pause and think of how many men and women were compelled to seek the aid of other hospitals solely on account of sin, whilst she argued that as Christians it was their duty to have compassion upon sinners, and to help them and to do what they could to mitigate their distress. It would be quite contrary to the spirit of their Divine Master if they said they would help only those who were blameless. She attached importance to limiting the aid to first cases, and asked those who failed to recognise that the mothers were often more sinned against than sinning to remember that the life and health of an innocent child were involved in the question.

The following vacancies are announced this week, the amount of salary in each case being given after the name of the institution :—

**Resident Medical Officers.**

South-Eastern Fever Hospital. Assistant Medical Officer.—Board, etc.
York Retreat. Junior Assistant Medical Officer.—£80, board, etc.
Huntingdon County Hospital. House Surgeon.—£60, board, etc.
Salop Foresters' Medical Association. Surgeon.—£160, house. etc.
Colchester Asylum for Idiots. Medical Attendant —£100, board, etc.

**Non-resident Medical Officers.**

Bristol Royal Infirmary. Surgeon and Assistant Surgeon.
Farringdon Dispensary. Honorary Physician.
Victoria Hospital for Children. Two Assistant Physicians to Out-patients.
Anderson's Medical College, Glasgow. Professor of Chemistry.
Dental Hospital of London. Dental Surgeon.

---

The Mendicity Society, Fisher Street, W.C., is issuing a special appeal, not only for funds, but against the giving of money to street beggars. The society provides its members with tickets which represent twopennyworth of food at any of Lockhart's Cocoa Rooms; the beggar who receives one of these tickets instead of coppers is usually a disgusted and disappointed man. If it is really food he wants of course he has now no longer need to hunger; but if, as is most probable, he sought the means of spending the night at a public-house, his plans are baulked. The society not only supplies these tickets, but it daily apprehends beggars and brings them before the magistrates. All this takes time and money. The books of the society chronicle the names of no less than 64,400 professional beggars, against some of whom fifty or sixty convictions have been obtained. We cannot too earnestly impress on our readers the necessity for aiding the society, both by subscribing to its funds, and by making it a rule never to give money relief to street beggars. Some of the most awful sufferings children have to endure are caused by the successful careers of many mendicants.

---

Annual reports, as a rule, are rather dull reading, but Dr. Moorhead, of Weymouth Royal Hospital, has managed to introduce a good deal of interest into his speech at the late meeting of subscribers to that institution. Touching lightly on the necessary facts and figures, he went on to describe the varieties of patients treated in the wards. He said : "In the surgical ward were several cases of an interesting character which are worthy of mention. That which constituted the *piece de resistance* of the year was one of malignant disease of the knee in which amputation was resorted to, at first apparently with success, but, unfortunately, followed some months afterwards by recurrence of the morbid development in the lungs, to which the poor fellow speedily succumbed. Another case was that of a packer in the employ of the Great Western Railway Company, who was knocked down by a heavy piece of metal falling upon him and inflicting severe scalp wounds. A man in the Royal Navy, a stoker in a torpedo boat, then anchored in Portland Roads, when on shore slipped and fell heavily upon the kerbstone, thereby sustaining concussion of the brain and other injuries. A cook belonging to the steam yacht *Jason* was admitted with severe septic inflammation of the hand arising from wounds by fish bones. The owner of the yacht gave a liberal donation to the hospital. A fireman of the s.s. *Winnebah*, bound from London to South Africa, was admitted for severe injury of the great toe inflicted by the wheel of the engine. The toe was so crushed as to have necessitated amputation. An able seaman of the s.s. *Cleveland*, bound from Hartlepool to Madras, was thrown down by an enormous wave which swept the deck and caused compound fracture of his left leg. These two sailors were Swedes. A seaman belonging to a ship trading with this port, who, unfortunately, while intoxicated a few weeks since, on trying to return on board fell into the harbour, and died some hours afterwards from failure of the heart's action, was also a patient.'

The new Royal Infirmary at Liverpool will be a magnificent building of grey brick, with terra-cotta dressings. The wards are arranged in six blocks, three on either side of this latter corridor, giving a total of 290 beds. Each of the thirty-two bed wards is 134 feet long and 28 feet wide, the height being 14 feet 6 inches. There are also four circular wards, each holding eighteen beds. Glazed bricks are largely used in the interior. The floors are of oak blocks laid on concrete, and the heating is by open small fireplaces in conjunction with steam. The radiators will be in the centre of the wards, supplied with fresh air from channels passing under the floors and open on both sides. The open fireplaces are placed against smoke and ventilating stacks also in the centre of the wards, the heat of the fires thus accelerating the removal of the vitiated air. The administrative block is nearly finished, and it is hoped the whole will be concluded this year. In the meantime, the temporary wards are doing their work well, and 1,661 in-patients were treated last year. There is still a debt of £2,000 waiting to be wiped out.

---

A very clever and comical series of sketches lately appeared in an American paper, which will probably do more for the cause of organized charity than a dozen solemn dissertations. The sketches were entitled "The Philanthropist and the Beggars: a lesson in multiplication." The first sketch showed a benevolent old gentleman, evidently on his way to business, bestowing a trifle on a blind beggar. The second sketch showed the same old gentleman, but beside the blind beggar stood another disreputable figure, bearing the placard "Please pity the Deef Mute." The third sketch again showed the old gentleman, but with an expression of surprise and horror on his face; for, beside the blind and the deaf mute, there now stood a "poor wider lady" and a "soldier's orphan boy." Presumably, however, the philanthropist managed to bestow charity on this assembly, for there was a fourth and last sketch showing a row of some ten beggars, all with extended hands waiting for the old gentleman. Poor Philanthropist! he could not face the destitution he had called up, and dropping his stick and losing his hat, is shown flying from the scene!

---

Sir James King made an excellent speech at the annual meeting of contributors to Glasgow Royal Infirmary. He first glanced at the past, and noted the slow growth towards perfection which was ever maintained. Then turning his attention to the present state of affairs, he said that during the past year no fewer than 46,000 persons had been treated in one form or another. As compared with the previous year, that showed an increase of 253, and there was a like increase in the number of cases that had been treated to a conclusion, so that they might say that the infirmary had been well occupied indeed during the past year, notwithstanding the additional accommodation which had been provided by hospitals outside. The mortality of the year, after eliminating the cases in which death resulted within 48 hours after admission, was exactly 6 per cent. Finances were not so satisfactory, and Sir James spoke with scorn of the "stereotyped guinea" which covers the charity of many who could well afford to be more liberal. Luckily the workmen are becoming larger contributors every year, and putting their richer brethren to shame. Mr. John Ure proposed, and Mr. Henderson seconded, that if the church collections continued so disgracefully small they should give up that source of income altogether. Mr. M'Ewen spoke on the subject of the increased use of the teaching facilities of the hospital which it was hoped to obtain by the Universities Bill. The Glasgow medical degrees were considered of less value than those of Edinburgh and London simply because there was not enough clinical demonstration of the excellent lectures. The proposed amendment of their charter would put this right, and then Glasgow Medical College should hold its own against all other colleges.

